# Broadening the selection criteria for Astronauts undertaking long–term space travel

**DOI:** 10.3389/fnume.2022.997718

**Published:** 2022-11-04

**Authors:** Hiroshi Yasuda, Lembit Sihver

**Affiliations:** ^1^ Research Institute for Radiation Biology and Medicine, Hiroshima University, Hiroshima, Japan; ^2^ Technische Universität Wien - Institute of Atomic and Subatomic Physics, Wien, Austria; ^3^ Department of Physics, Chalmers University of Technology, Gothenburg, Sweden; ^4^ Department of Radiation Dosimetry, Nuclear Physics Institute of the CAS, Prague, Czech Republic

**Keywords:** astronaut, space travel, radiation exposure, health risk, deep space mission

One of the major concerns in future deep space missions to the moon and Mars is the increased radiological risk of astronauts. They will be exposed to enhanced levels of ionizing radiation from natural sources, such as galactic cosmic radiation, radiation from the Sun including high-energy charged particles at solar particle events (SPEs), and radiation belts surrounding the Earth (1, 2). The accumulated radiation dose over a long-term mission to Mars is estimated to reach 1 Sv or more, depending on duration, shielding and time in the solar cycle (3). Although it is a rare event, SPE particles could further increase their doses to a serious level up to as high as 10 Gy (4), which is far beyond the dose limit for radiation workers (5), and could induce severe acute deterministic effects such as deterioration of blood-forming function (6), reproductive potential (7), cataract (8) and even death by acute radiation syndrome.

With these concerns, space agencies such as the National Aeronautics and Space Administration (NASA) of the USA and Japan Aerospace Exploration Agency (JAXA) have developed dose-limitation criteria for controlling the space radiation exposure of astronauts below an acceptable level. Table 1 shows the previous career dose limits for NASA (1) and the current limits for JAXA (9) astronauts involved in low-Earth-orbit missions. The limits of NASA were determined to constrain the increasing cancer risk incurred by an astronaut to 3%; more precisely, the limits for NASA astronauts were not to exceed 3% risk of exposure-induced death from fatal cancers at a 95% confidence level based on a statistical assessment of the uncertainties in the risk projections (10). As the unit–dose cancer risk generally increases with age (5, 11, 12), the dose limit became higher for older astronauts than that for young ones. In addition, at the same age range, a limit value for female astronauts was higher than that for male ones, reflecting the fact that the breast has a notably higher radiosensitivity (5, 11, 12).

**Table 1:** Previous career dose limits for the astronauts of NASA (USA) (1) and current limits for JAXA (Japan) (9) involved in low-earth-orbit space missions.

Space agency (country)	Male (Sv)	Female (Sv)
NASA (USA)	1.5 Sv for 25 y	1.0 Sv for 25 y
	2.5 Sv for 35 y	1.75 Sv for 35 y
	3.25 Sv for 45 y	2.5 Sv for 45 y
	4.0 Sv for 55 y	3.0 Sv for 55 y
JAXA (Japan)	0.6 Sv for 27 y	0.5 Sv for 27 y
	0.7 Sv for 31 y	0.6 Sv for 31 y
	0.8 Sv for 36 y	0.65 Sv for 36 y
	0.95 Sv for 41 y	0.75 Sv for 41 y
	1.0 Sv for 46 y	0.8 Sv for 46 y

The limit value is given as effective dose for the age of the first flight exposure.

As inferred from the values in Table 1, these dose-limitation criteria allowed older male astronauts to have more opportunities of space travel than young or female astronauts, which could be regarded as a problem of inequality. Then, the National Academy of Sciences (NAS) in United States recently made a recommendation of applying a 600 mSv age and gender independent career limit of effective dose based on a median estimate to reach 3% cancer fatality for 35-year-old females (13), withdrawing the age and gender speciﬁc limits. This recommendation is expected to allow equivalent ﬂight opportunities for all male/female astronauts of different ages (13). Following this recommendation of NAS, NASA updated the standards for crew health in 2022 (14). In the new standards, the total career effective dose of an astronaut due to space flight radiation exposure shall be less than 600 mSv and this limit is universal for all ages and sexes. It is also required that radiation exposure from all sources below the limit shall be further minimized following the principle of “as low as reasonably achievable (ALARA)”.

This concept has been criticized by some researchers for the reason that it could have negative impacts on crew health and safety and violate the principles of radiological protection (15). It is worried also that such a lower annual dose limit would make a Mars mission unfeasible since the cumulative dose in one mission is expected to far exceed 600 mSv. While, recognizing the fact that the risk of radiation-induced cancer can considerably change among individuals and also under different radiation dose rates, the National Aeronautics and Space Administration (NASA) had requested National Council on Radiation Protection and Measurements (NCRP) in United States to evaluate the risk of radiation-induced lung cancer in populations exposed to chronic or fractionated radiation to learn whether differences exist when exposures occur gradually over years contrasted with the acute exposure received by the Japanese atomic-bomb survivors. In response to the request from NASA, NCRP launched a scientific committee and have been working to prepare a commentary (16) on this issue with accompanying recommendations for NASA.

Considering such a fluid situation on the radiological protection criteria for astronauts, the authors like to present here a different viewpoint which might mitigate the ongoing discussion on radiological protection of astronauts. Apart from the possible ethical issue of discriminating people by age or gender, it is known that elderly people have generally more health problems related to aging. Any person inevitably becomes vulnerable with age through various types of deteriorative changes due to many causes (17, 18), although the pace of aging varies among individuals (19, 20). Some of the typical aging symptoms that could be commonly experienced before age 65 years (general retirement age in many countries) are as follows:

- Loss of muscle mass and strength (21)
- Weakening and embrittlement of bones (osteoporosis) (22)
- Loss of arterial elasticity (atherosclerosis) and other cardiovascular changes (23)
- Difficulty in focusing eyes on close objects (presbyopia)
- Lowering hearing ability of ears (presbycusis)
- Cardiovascular diseases
- Menopause associated with hot flash, disruption in sleep, subsequent osteoporosis, etc.

In addition, risks for cognitive impairment (dementia, Alzheimer's disease, Parkinson's disease, etc.) (24), cataracts (8), and carcinogenesis (12, 23) increase with age. With advancing age, individuals also tend to have difficulty coping with various stresses such as strenuous exercise and environmental changes; and those stresses tend to cause functional deterioration of some of their organs such as the heart, urinary organs, and brain. The senescence-associated health deterioration can be different between male and female people, as women have some biological advantage related to their ability to bear a child and the physiological systems that permit pregnancy (25).

For preventing the occurrence of a serious problem caused by the aging of astronauts in long-term deep-space mission, it is desirable to carefully consider all possible age- and gender-related deterioration of health when selecting the astronauts who will take up difficult tasks during a long space mission for up to few years. While young astronauts are generally tough and swiftly acting, experienced older astronauts are more knowledgeable and prudent, which would make them more reliable when facing unexpected troubles. The current criteria of NASA on crew health management (14) does not clearly indicate how to evaluate and balance the unique competences of individual astronauts who will work together for a long period in the same mission. According to these facts, the authors propose to deal with the age- and gender-dependent radiation sensitivity as one of the major qualifications required for astronauts involved in a deep space mission.

**Figure 1:**
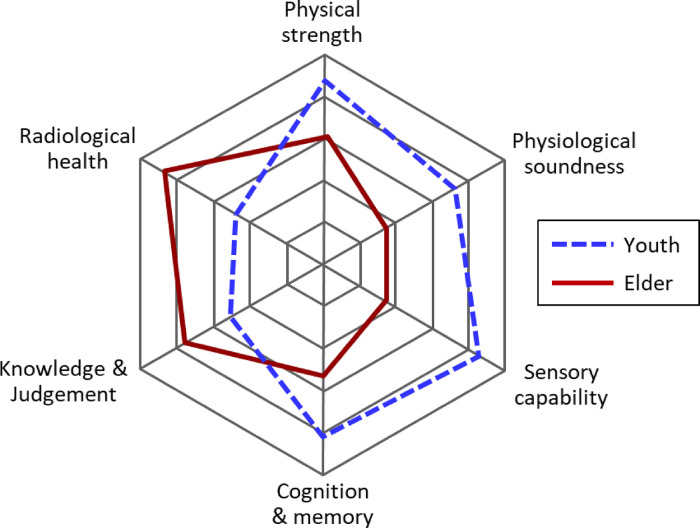
Example of scoring in regard to major requirements on qualification of astronauts and assumed typical scores of young and old candidates. Radiological health (i.e., the inverse of radiosensitivity) should be one of the requirements for astronauts involved in long-term space missions.

The conceptual basis of this idea can be illustrated with a radar chart as shown in Figure 1. This chart has six axes of major requirements on qualifications of astronauts with two example patterns of typical scores of young and older astronauts. The requirements assumed here are (1) physical strength related to muscles, bones, and cartilage; (2) physiological soundness related to cardiovascular, renal, digestive, respiratory, and immune systems; (3) sensory capability related to perceptions with eyes, ears, nose, and nerves; (4) cognition and memory related to the neuroimaging functions mainly controlled by brain; (5) knowledge and judgement supported by acquired intellectual base and experience; and (6) radiological health which could be quantified as an inverse quantity of radiation sensitivity regarding carcinogenesis. Older astronauts could have higher scores on the knowledge, judgement and radiological health, while their scores on physical strength, physiological soundness, sensory capability, and cognitive functions would be lower.

For the success of future deep-space missions, it will be crucial to carefully select healthy astronauts who can show strength against not only psychosomatic stress including radiation-induced cancers, but also the inevitable aging effects during a long traveling period of up to few years; possible appearance of different-quality age-associated symptoms should be projected in the process of crew selection. In this sense, a routine health surveillance programme based on the general principles of occupational health will take a vital role for assessing the initial and continuing fitness of the astronauts for achieving their intended tasks as a team in a specific space mission. With these efforts, it is expected that a well-balanced team of male and female astronauts covering a broad range of age will be formed, so that the scores regarding all requirements as shown in Figure 1 could be maximized as a whole.

In conclusion, the diversity in formation of a team of astronauts is preferably to be pursued for successful deep space missions in the future. For this, further studies for overcoming various aging-related health issues are needed to smash the current highest score.
